# A Decade of Evidence of Sorghum Potential in the Development of Novel Food Products: Insights from a Bibliometric Analysis

**DOI:** 10.3390/foods12203790

**Published:** 2023-10-16

**Authors:** Etiene Valéria Aguiar, Fernanda Garcia Santos, Valéria Aparecida Vieira Queiroz, Vanessa Dias Capriles

**Affiliations:** 1 Laboratory of Food Technology and Nutrition, Department of Biosciences, Institute of Health and Society, Campus Baixada Santista, Federal University of São Paulo (UNIFESP), Rua Silva Jardim, 136, Santos 11015-020, SP, Brazil; etiene.aguiar@unifesp.br (E.V.A.); fg.santos@unifesp.br (F.G.S.); 2 Brazilian Agricultural Research Corporation, Embrapa Milho e Sorgo, Rodovia MG 424, km 65, Sete Lagoas 35701-970, MG, Brazil; valeria.vieira@embrapa.br

**Keywords:** gluten-free, *Sorghum bicolor* L. *Moench*, whole grain, food processing, bioactive compounds

## Abstract

Due to the increasing interest in sorghum for human nutrition, recent literature reviews highlight its nutrient and bioactive contents, potential health benefits and its ‘gluten-free’ feature. Moreover, a current view of research advances on sorghum-based food products is needed to help both food scientists and industry identify current trends and forward-looking approaches. Studies on homemade processing are still scarce. Thus, this review aimed to provide the latest information regarding the use of sorghum to develop ready-to-eat products or food ingredients based on studies published in the last decade (2012–2022), which then guided discussions on recent advances and prospects. The articles were identified by searching the Elsevier Scopus database. Sorghum has great potential as a functional and sustainable food that can be used in daily meals as a substitute for common cereals like wheat, rice and corn. The studies in the review show that it is possible to process sorghum in a wide variety of ways to obtain ready-to-eat products and ingredients for food products and preparations, such as popping, lamination, extrusion and wet cooking. The studies also show promising approaches to use sorghum in acceptable and nutrient-dense bakery and pasta products, highlighting their gluten-free versions. However, more efforts to make these novel food products available to consumers should be made.

## 1. Introduction

Sorghum (*Sorghum bicolor* L. *Moench*) is the fifth most produced cereal and, in most countries, is mainly used as animal feed. However, due to the interesting nutritional content of this cereal, it can be successfully used to improve the nutritional content of starch-based products that are commonly developed with non-wholemeal flours like wheat, rice and corn [1,2].

The increase in whole grain consumption has been correlated to the decrease in the incidence of non-communicable chronic diseases, a fact that has increasingly aroused the interest of researchers in the study of cereals such as sorghum [3,4]. Studies indicate sorghum to diversify the diet and promote human health through its nutritional composition and potential as a functional food. Among the physiological effects already investigated, sorghum shows good antioxidant activity, lower glycemic response and greater benefits to the intestinal microbiota when compared to other cereal grains [5,6,7,8]. However, despite the human health benefits of sorghum consumption, its cultivation in some countries is still primarily intended for animal feed [9].

Over the last ten years, there has been growing interest among researchers in the health benefits of sorghum consumption [10,11,12] and the use of this grain to develop healthier food products [13,14,15]. Sorghum flour usually presents a high content of dietary fiber, fat and protein in addition to some micronutrients and several bioactive compounds that can contribute to improving the nutritional quality of cereal-based products for human consumption [11,13]. Another advantage of sorghum is that it is gluten-free (GF), making it an ingredient of great interest for those who seek to improve the quality of GF foods. These GF products often have poor nutritional quality since they are neither enriched nor fortified. They also present low technological and sensory quality and do not look or taste good [16,17].

Some researchers evidence the high glycemic index of wheat-based and GF bakery products, and so, the substitution of common cereals with sorghum flour can contribute to the development of products with a lower glycemic index due to the dietary fiber and resistant starch contents of sorghum [6,12]. Also, some genotypes contain tannins in the composition, which have already shown a positive relationship with a decrease in the glycemic rate [10,18].

Due to the increasing interest in sorghum for human nutrition, recent literature reviews highlight its nutrient and bioactive content as well as its potential health benefits [6,10,12] and focus on the milling, malting, fermentation and thermal processing of sorghum to be consumed or used as a food ingredient [11]. Moreover, a current view of recent research into sorghum-based food products is needed to help both food scientists and industry identify current trends and forward-looking approaches as well as to design strategies to diminish the challenges regarding the use of sorghum for human nutrition, including the commercialization of sorghum-based food products.

Thus, this review aimed to provide the latest information regarding the use of sorghum to develop food products for human consumption, highlighting its potential for GF foodstuff and diet, based on studies published in the last decade, which then guided discussions on recent advances and prospects.

## 2. Research Methodology

**Figure 1:**
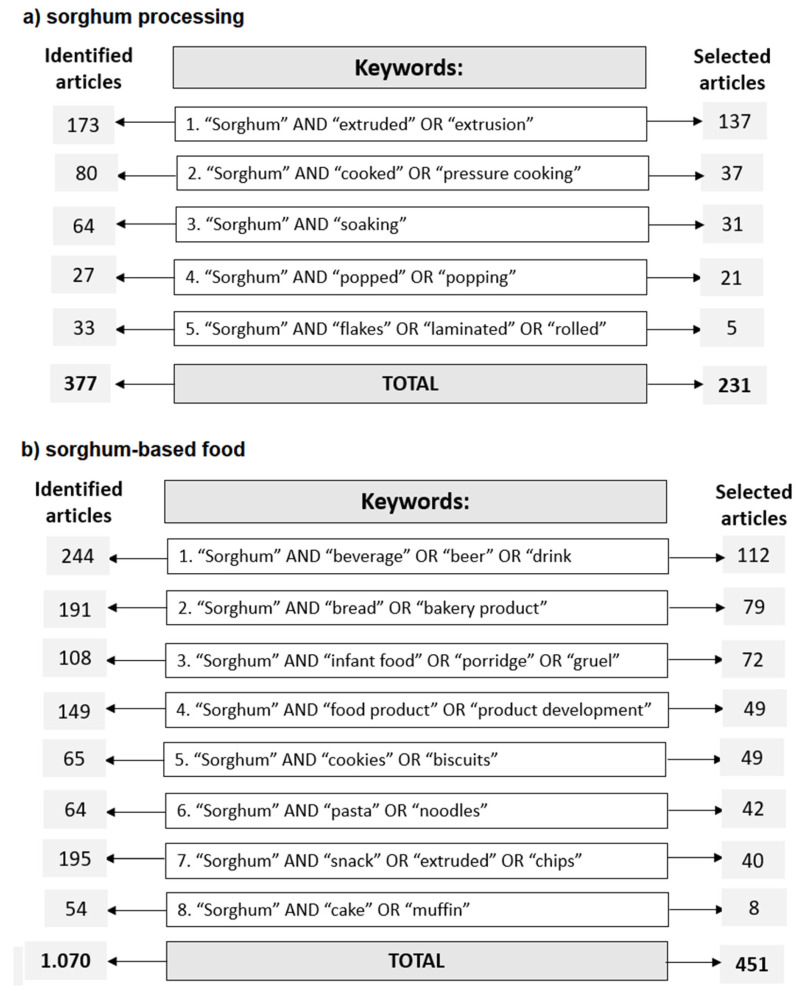
Flow charts pertaining to the selection process of papers on Elsevier Scopus database, summarizing the obtained results of the literature review.

The Elsevier *Scopus* database was used to select the studies about sorghum processing and sorghum-based food products. For this search, the specific terms presented in Figure 1 were used. Original articles published in the last ten years (2012–2022) with no restrictions regarding language that reported sorghum processing or sorghum-based products were included, while other papers that were not pertinent to sorghum processing or products were excluded. The studies were selected by reading the title and abstract and by a full-text reading and exclusion of the duplicated selected papers.

All items from the citation information (e.g., authors, document title, year and citation count), bibliographical information (e.g., affiliations), abstract and keywords using the search terms were selected and exported in CSV format and used to construct a keyword co-occurrence and a countries co-authorship map using the VOSviewer software v1.6.17 (Leiden University, Leiden, The Netherlands).

The selected papers were grouped by type of product during the period 2012–2022 to verify advances in the use of sorghum in products for human consumption.

## 3. Main Findings

### 3.1. Sorghum Processing

**Figure 2:**
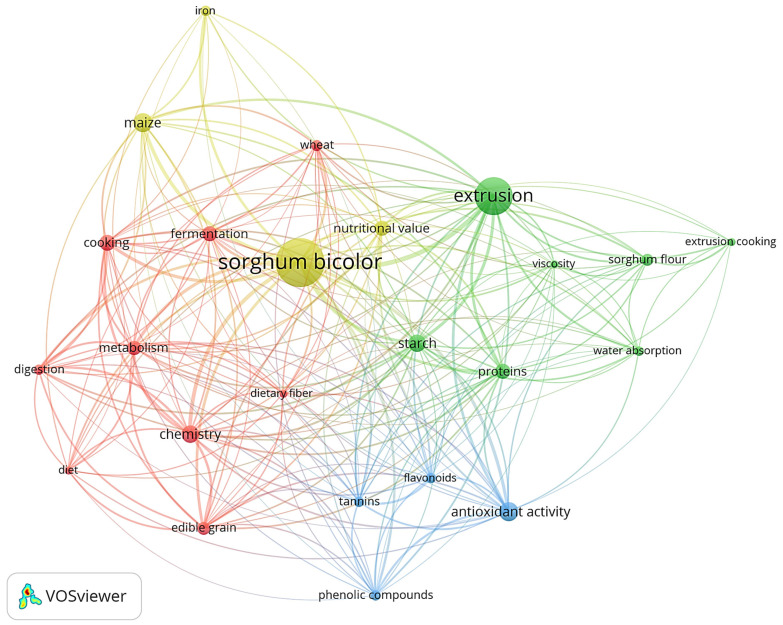
Keyword co-occurrence network from articles on sorghum thermal processing. Size of the bubbles represents more frequently used terms, while thicker connectors correspond to more frequent co-occurrence of terms. Source: Scopus database (2012–2022). A minimum of 8 occurrences was adopted to integrate keywords analysis using the VOSviewer software.

The sorghum grains can be processed in various ways to enable their consumption as human food, and Figure 2 shows the search topics that studied these processes, presenting the 37 most used keywords in the original articles regarding sorghum-based products for the period 2012–2022. For this bibliometric map, 229 original studies selected from the Scopus database were used, based on the specified search terms described in Figure 1a.

Figure 2 is arranged into four clusters. The largest one (in red) has 14 items, which are related to sorghum use in food processing with the objective of fortifying the products. This cluster shows the fermentation and germination processes, evaluating the process influence on the phytic acid and mineral content. The second cluster (in green) has 11 items; this cluster includes the studies about the extrusion process, mainly the optimal processing characteristics, such as water and temperature adjustments, and checking the flour gelatinization and viscosity as well as the overall acceptability of the final products. The third cluster (in blue) contains eight items that address the cooking of the sorghum grains, verifying the functional properties like the antioxidant capacity and analyzing the presence of phenolic compounds like flavonoids and tannins. The fourth cluster (in yellow) has four items, which are related to the dietary fiber, metabolism and digestibility studies that are principally tested using animal models.

Figure 1 and Figure 2 indicate that studies with homemade processing are still scarce, and the potential of sorghum as a functional and sustainable food that can be used in daily meals as a substitute to common cereals such as rice and corn needs further investigation [19,20].

Sorghum is a cereal with several nutritional advantages since it is rich in phenolic compounds and anthocyanins and has a high antioxidant capacity [9]. Research shows the domestic processing of sorghum grains as beneficial because these processes can diminish the tannins and phytic acid content in the sorghum grain. However, recent research relates the benefits of the high tannins in sorghum with the advantages of this cereal in developing food products with a higher antioxidant capacity. More studies are needed to better elucidate the process for the various sorghum genotypes based on the nutritional quality as well as the viability in terms of grain yield, practicality in homemade and industrial methods and consumer acceptability [3,4]. New studies need to investigate the processes with various sorghum genotypes to know the impact on each grain. It is also necessary that studies include a comparison of the processed sorghum with the raw version and with common cereals, being that this is essential to elucidate the sorghum potential to substitute the usual consumed cereals and to evidence the advantages and disadvantages against each processing method tested.

#### 3.1.1. Soaking

Soaking is a common household method used principally to reduce the cooking time and to improve the nutritional quality of legumes, as it can reduce the phytate content and enhance the bioaccessibility [21].

Looking at the studies that used soaking with this ancestral cereal, it is clear that few studies seeked to evaluate the characteristics of this cereal for human consumption.

For human food, soaking is very common for grain germination and in beverage production [22,23]. The soaking process has been used in some research to reduce the anti-nutritional components of sorghum grains, such as tannins, oxalate and phytic acid, intending to improve the digestibility and the bioacessibility [24,25].

Keyata et al. [26] evaluated the composition, the protein digestibility and mineral bioavailability of sorghum grains after different kinds of processing (control, washing, soaking, malting and germination), observing that the soaking process reduced the antinutrient factors and improved the protein digestibility and mineral content and bioavailability. However, in Afify et al. [24] and Afify et al. [25], there were reduced vitamin E, β-carotene, total phenols, total flavonoids, tannins, phenolic acid and flavonoid contents as well as lower antioxidant activity when including the soaking process to prepare the grain. Kruger, Oelofse and Taylor [27] evaluated the mineral availability in soaked sorghum grains, observing a reduced content of soluble fibers, and the authors emphasize that, even with the reduction in antinutritional factors, soaking seems not to be a viable method to improve the nutritional quality of sorghum since it reduced the minerals available in the grain.

The studies that evaluated soaked sorghum showed nutritional benefits of the soaking stage in the germination, fermentation and toasting processes [24,25,28,29], which are common to Asian people. Further studies are needed to assess the advantages of including the soaking stage in processes common to other cultures, such as cooking and popping, to verify the practicality in preparation, the nutritional benefits and the sensory acceptance by the consumers. This can contribute not only to elucidating the best way to prepare sorghum grain but also to show its potential for daily consumption in human nutrition.

#### 3.1.2. Wet Cooking: Common and Pressure Method

Sorghum is usually cooked before eating. The Scopus search shows the most common cooking process for sorghum uses the flour to make porridge. Pressure cooking is a household process that shortens cooking time. Some studies show that pressure cooking maintains more bioactive and phenolic compounds and, also, a higher antioxidant activity than normal cooking [9,30].

In the search for studies that used a pressure cooker with sorghum grains to pressure cook, 31 articles analyzed sorghum using the cooking process of which just 17 studies were developed using sorghum grains with the other 14 studies using sorghum flour.

Most of the studies cooked the sorghum grains or flour in water to verify the nutritional quality, nutrient bioaccessibility and, also, to analyze the protein and starch digestion with no sensory evaluation of the products. Only two studies were found that used pressure cooking that also verified the sensory acceptance. Tran and Chambers [31] looked at five products made with commercially available sorghum using different processes (cooked grains, porridge, cookies, muffins and extruded puffed snacks) and, through the sensory analyses, verified that cooked sorghum grains and the cookies made with sorghum flour presented distinct flavor attributes, being recognized as the samples with the greatest flavor of the original grain. Hicks-Roof et al. [32] tested cooked grains with college students and concluded from the comparative sensory analyses that the cooked sorghum was as well accepted as cooked refined rice, being a good option to replace the refined cereals and contribute to an increase in whole grain consumption.

More studies could evaluate different cooking times, aiming to define the ideal proportion between water, grain and cooking time and also analyzing the influence of cooking time on the nutritional content and consumer acceptance.

#### 3.1.3. Popping

The popping process occurs when the moisture content in the center of the endosperm vaporizes and raises the pressure in the endosperm, causing it to rupture and burst apart the outer endosperm [33]. There were 14 studies that reported the use of the popping process, using, namely, a popcorn maker (n = 6), hot air chamber (n = 4), microwave (n = 3) and an adapted fluidized bed dryer (n = 1). Most of the studies analyzed cited a moisture adjustment that ranged from 11 to 16% before submitting the grains to the popping process with the aim of improving the yield and volume of the popped grains.

Most of the studies are far from the domestic reality because they adjust the moisture of the grains or use equipment not common in home kitchens. Studies are needed that prepare the popped sorghum through a simple method that can be used at home, identifying the sorghum genotypes that can replace corn in the popping process and indicating those that present good volume and yield with low numbers of unpopped grains and, also, that present a sensory acceptance similar to popcorn.

Only two studies included a sensory analysis of popped sorghum, both using the 9-point hedonic scale to verify the appearance, taste and overall acceptance. Both studies used oil and salt to prepare the samples and reported good acceptance of the popped grains. A comparative study to look at the sensory differences between sorghum genotypes and common cereal grains, such as rice and corn, is needed to demonstrate the viability of using sorghum as a substitute.

#### 3.1.4. Lamination

The lamination process is based on the lamination of cereal grains with a suitable mill, being widely used in oat flake production, while for sorghum grains, it is still little used. HameedaBanu et al. [34] used the lamination process to make sorghum flakes. The authors used a fluidized bed roaster, which is a method that can be reproduced in industry, contributing to diversification of commercial cereal-based food products. These flakes are a good breakfast option as a substitute for the more usual breakfast cereals made with corn, wheat or oats.

There were only a few articles that used lamination to produce sorghum flakes. There are some studies that produced flakes using sorghum flour, but these flakes were made by mixing the sorghum flour with other ingredients, producing a dough that was passed through a dough cylinder. This was then cut into circular shapes, characteristic of breakfast cereals, and toasted. These studies made use of an extruder with the flour [35] or with the flake dough [36].

To date, no study has been found with sorghum lamination for flakes, using a homemade method, that also has a sensory analysis of the final product.

#### 3.1.5. Extrusion

Extrusion cooking is usually used to modify the characteristics of raw materials and improve the flavor of the final product. Okafor and Falade [37] cited important advantages of submitting cereals to the extrusion process in addition to the enhanced palatability, such as the production yield, which can help reduce losses related to the underutilization of these crops.

Espinosa-Ramírez et al. [38] reported that some whole grain flour presented techno-functional properties comparable to those extruded made with refined rice flour, such as the extruded made with whole sorghum flour that has better nutritional characteristics than the refined rice flour; the extruded sorghum has a similar energy content to the extruded rice but with an improved phenolic compound content and increased antioxidant activity due to the nutritional characteristics of sorghum, demonstrating the potential of this ancestral cereal as an interesting ingredient for the development of new functional whole-extruded products.

The extrusion process is extensively used by the industry to develop some kinds of food products, like ready-to-eat snacks, dehydrated instant food, breakfast cereals, beverages and baked products [39]. However, most of these products are mainly composed of carbohydrates, since the main ingredients are refined cereals and/or starch with low levels of protein. The use of whole grain flours, such as sorghum, could improve the nutritional quality of these extruded products [37].

There were 139 studies (Figure 1) that used extrusion with sorghum with grains rather than flour being studied more. The studies with extruded sorghum reported the ideal process conditions and nutritional content and included studies about digestibility. The extruded products using sorghum flour in the composition presented a good acceptance, being similar to the extruded products made with refined flours (wheat or rice flour). However, it is important to highlight that less than 19% of the selected studies used sensory analysis, which is of extreme importance in showing the real acceptance of these sorghum-based products.

### 3.2. Sorghum-Based Food Products

**Figure 3:**
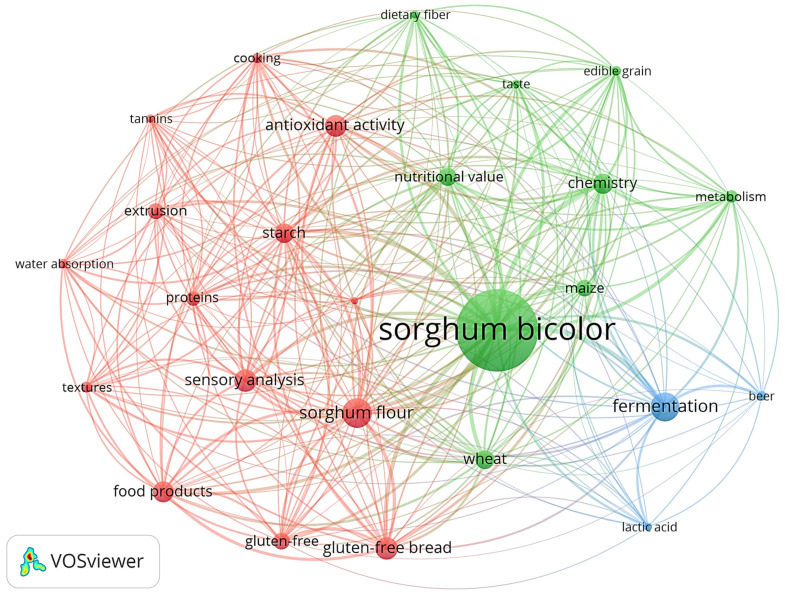
Keyword co-occurrence network from original articles regarding sorghum-based products. Size of the bubbles represents more frequently used terms, while thicker connectors correspond to more frequent co-occurrence of terms. Source: Scopus database (2012–2022). A minimum of 20 occurrences was adopted to integrate keywords analysis using the VOSviewer software.

Based on the inclusion criteria, 451 original articles were selected (Figure 1b) and used to create the bibliometric map based on research about sorghum-based food products (Figure 3).

Figure 3 shows the 26 most used keywords in the original articles regarding sorghum-based products arranged into three clusters (Figure 3). The red cluster is composed of 14 keywords that are related to the development of new food products and draw attention mainly to GF products, especially the GF bread developed with sorghum flour, analyzing product characteristics through sensory analysis, nutrient content and antioxidant activity. The extrusion and cooking processes are also included in this cluster. The green cluster that contains 10 items represents a common research approach to sorghum flour in combination with common ingredients, such as maize or wheat flour, showing the differences in nutritional value, metabolism and the taste of these products. The blue cluster (three items) illustrates the studies about the fermentation process of lactic acid and its relationship with beer development. The figure lays out the research about sorghum used to developed GF products for human consumption, showing that this ancestral cereal is a good option to substitute common cereals, like wheat, rice and corn.

**Figure 4:**
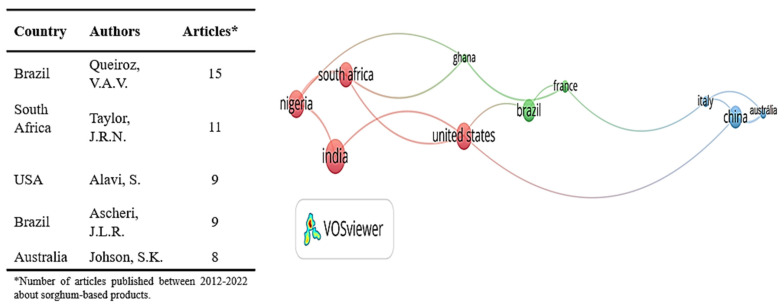
Country co-occurrence network and main authors from original articles regarding sorghum-based products. Size of the bubbles represents more frequently used terms, while thicker connectors correspond to more frequent co-occurrence of terms. Source: Scopus database (2012–2022). A total of 14 articles was adopted as the minimum number of documents to include a country in the analysis using the VOSviewer software.

The 451 original articles were also analyzed according to the authors and country co-occurrence as shown in Figure 4. In descending order, India (12.6%), Nigeria (10%), the United States (9.7%), South Africa (9%), Brazil (8%) and China (8%) are the main countries of origin of the selected studies, while for the authors, the main researchers are from institutes in Brazil, South Africa and the United States. Among them, there is scientific cooperation between the United States and South Africa or Brazil; collaboration between Brazil and South Africa should be further stimulated.

The African continent is responsible for more than 22% of sorghum research, being the main continent to contribute with studies about sorghum for human nutrition. However, India is the country that produced the most research about sorghum (12.6%), while France, South Africa and the United States (USA), compared with all 84 countries that produced some of the selected articles of sorghum, were regarded as the leaders for research collaboration with other countries. This scientific cooperation between the different countries is essential to investigate all sorghum genotypes and traditional and novel food processes and to contribute to a greater inclusion of sorghum in human food.

Even though the sorghum grain is one of the main grain crops planted in Brazil, and Brazilian researchers are responsible for the largest number of studies on sorghum-based products for human consumption, sorghum continues to be produced mainly for animal feed, while in Asian and African countries, its cultivation is largely intended for human consumption, playing an important role in the food security of these populations and showing the potential of the grain for use in food for human consumption [1,2,40]. Until now, no research collaboration between Brazil and South Africa or India was noticed. This scientific cooperation is important to provide a better knowledge of traditional African and Asian uses of sorghum, allowing for its adaptation to the western diet as well as different countries’ and regions’ food products and preparations.

**Figure 5:**
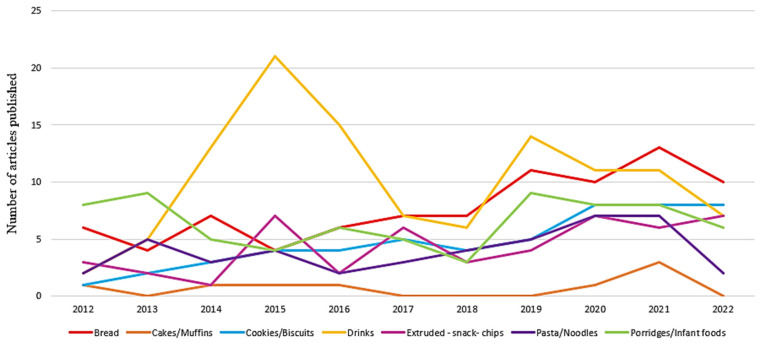
Comparison of total scientific production of original articles about different sorghum-based products in terms of title, abstract and keywords registered in Elsevier Scopus database (2012–2022).

Sorghum is indicated as a cereal with high potential to be used in the development of various starchy-based food products for human consumption, such as bakery products, extruded products, beverages and porridges, being very appropriate for the development of GF products [13,14,15]. Figure 5 shows the total scientific production of original articles about different sorghum-based products (2012–2022), showing the categories of these products.

Detailed information about sorghum-based products is provided in the next sections and Table 1.

#### 3.2.1. Beverages and Syrup

Beverages used to be the most researched sorghum-based products with beer as the main type of drink as it is commonly produced and consumed in Africa and China. Davana and Revanna [22] developed beers using different proportions of sorghum with barley (40%, 60% and 100% of each cereal), and the 60% sorghum beer received the highest score based on the mouth feel. From the organoleptic evaluation, as observed in Table 1, the sorghum beer was comparable to commercial beer made with barley, demonstrating the potential of sorghum as an alternative cereal in beer production, especially in GF beers that benefit people who do not tolerate gluten, and having an advantage from the economic point of view due to the lower planting cost of sorghum.

Besides the beer, sorghum is also interesting for juice development. Sharma et al. [41] developed a juice using sweet sorghum. The juice was extracted using a mechanical extractor, sterilized by heating at 90 °C for 15 min and then centrifugated for 10 min at 6000 RPM to remove the insoluble particles. [41] converted the fermentable sugars present in the juice into functional carbohydrate molecules, producing a well-accepted functional beverage that was rich in prebiotic oligosaccharides, which can contribute to gut health. This demonstrates the potential of sweet sorghum juice as a low-cost raw material for producing new beverages with functional appeal.

Cséfalvay and Bakacsi [54] and Mazumdar et al. [55] investigated the use of sweet-sorghum juice to produce a syrup with improved nutritional quality that was also well accepted by the consumers. Sorghum syrup is an interesting product because it can be used by the industry as an alternative sugar to sweeten different food products. The cited authors drew attention to the fact that the syrup, which is made from the sweet-sorghum juice, has the potential to be used in developing commercial beverages since they can be further fortified or blended with other fruit juices or concentrates. They can also be with protein concentrates or other beverages, allowing for the development of products with an improved nutritional quality and with good acceptance by the nutraceutical segment due to the health benefits provided by the sorghum [10,11,12].

#### 3.2.2. Porridge

Figure 4 shows that, like beer, sorghum porridge used to be the most developed product in research. Rashwan et al. [15] reviewed various technological processing methods, such as soaking, germination, fermentation, thermal processes and irradiation that could contribute to improving the nutritional quality of sorghum porridges, and the authors indicated fermentation as the principal treatment to benefit the nutritional value of sorghum-based products, using some other combined treatments such as soaking, germination and nixtamalization (soaking and cooking in limewater), demonstrating the contribution to producing sorghum-based foods with a higher nutritional value.

Makame et al. [42] investigated the oral texture properties of some complementary indigenous porridges, indicating the sorghum-based porridge as “not easy to swallow even with low solids content”, presenting a viscosity higher than that indicated for children up to 3 years old. The difficulty in swallowing the porridge can limit food and nutrient intake, perpetuating protein and energy malnutrition in infants that rely on these types of food. These data call attention to the necessity of research involving different techniques to reduce the viscosity of sorghum-based porridges, which may contribute to easier consumption by children under 3 years of age.

Adebowale et al. [43] microwaved sorghum grains, which resulted in a lower viscosity of the porridges made, with these treated grains being more adequate for infant feeding. The authors highlight the necessity to evaluate the porridge acceptance with children, since they have different chewing, bolus formation and swallowing process from adults. However, the economic viability of microwave treatment needs to be evaluated to validate its applicability in an industrial production line.

#### 3.2.3. Bakery Products

Since 2014, there has been an increase in research about sorghum-based bakery product development, mainly GF bread. Aguiar et al. [17] showed that, during the last ten years, commercial gluten containing (GC) (n = 7122) and GF (n = 3153) food products have been investigated and compared worldwide. The label evaluation of GF bread (n = 935) reveals that this product is composed of multiple raw ingredients and additives, resulting in a high variability in the carbohydrate (14 to 84%), fat (1 to 19%), protein (0 to 11%), sugar (0 to 24%) and dietary fiber (0 to 17%) contents. These GF products continue to be recognized as high in fat, low in protein and, in some cases, low in dietary fiber. The use of sorghum to produce GF breads can improve not just the nutritional content but also the physical and sensory properties, as reported by some authors like Centeno et al. [14] who developed well-accepted GF bread using up to 75% of white sorghum flour (BRS501 genotype without tannins) combined with 25% potato starch or using 100% of bronze sorghum flour (BRS332 genotype containing tannins).

Khoddami et al. [11] reported lower sensory acceptance of sorghum-based products when compared to other cereal products. In fact, as pointed out by de Oliveira et al. [56] who investigated the acceptance of GF bread developed with different sorghum types and flours, the presence of tannins and other phenolics in sorghum-based bread may contribute to a negative effect on the acceptance. However, the authors draw attention to the need to indicate the most suitable product for each sorghum hybrid, since GF bread developed with the red sorghum BRS 332 flour was well-accepted.

For the development of cakes with no color impact, the sorghum with the white pericarp can easily be used to substitute for flours like wheat and rice, while for products that contain chocolate, it also possible to use the brown and red genotypes, which contribute to a darker product that has a positive impact on the physical appearance [14,57,58].

Sorghum contributes to good physical characteristics of cakes and also provides an improvement in the nutritional quality and high acceptance from the consumers, indicating the cakes made with sorghum are very similar in terms of flavor, odor, texture and color to those traditional products made with wheat or rice flour [45]. Cayres et al. [59] developed GF cakes that contained 87.8% red sorghum wholegrain flour (flour basis), demonstrating that sorghum can be used as the main ingredient for this food product. The authors draw attention to the fact that, when questioned, the Brazilian consumers stated that they did not know about sorghum, so its use has the potential to be offered as a novelty on the market.

Cookies and biscuits are products that can also benefit when developed with sorghum because both the GC and the GF versions can use this ingredient to improve their nutritional quality. Yu et al. [57] developed biscuits that contained wheat flour (WF) enriched with whole sorghum flour and extruded sorghum flour (ESF). The use of 80% of ESF with WF biscuits was indicated to improve the resistant starch content of these products with this sample obtaining a higher sensory acceptance than the control sample prepared with 100% WF. Cervini et al. [60] developed GF biscuits enriched with a novel resistant starch ingredient obtained from annealed white sorghum starch. The use of resistant starch is interesting because the partial substitution of flours like the WF for this ingredient can be a great alternative to the commercially developed products containing starches with low digestible properties, containing a higher fiber content. However, even with a positive effect on the nutritional quality, the biscuits made with the resistant starch presented a low texture score and low overall acceptance, being harder when compared to the control sample made with 100% of commercial GF flour mixture.

One of the principal challenges in the production of GF foods is the low acceptance of texture because the lack of gluten directly impacts the hardness. Consequently, products developed for their health appeal need to focus on the sensory acceptance since, despite so much research about GF products, they are still not very satisfactory in terms of sensory characteristics, mainly flavor and texture, for both celiac and non-celiac GF consumers [16,61].

#### 3.2.4. Pasta Products

Regarding pasta products, the literature reports the use of sorghum in high quantities without a negative impact on the sensory acceptance. Johnson et al. [58] found that the use of up to 75% of black sorghum flour combined with WF can be used to produce noodles with improved nutritional quality, increasing the total polyphenolic content and presenting a higher antioxidant capacity as well as a sensory acceptance comparable to the standard sample produced with 100% WF. De Oliveira et al. [56] investigated the use of sorghum to develop GF pasta using 24.4% white (commercial and CMSXS 180), red (BRS 330 and BRS 332) or brown (BRS 305 and 1167048) sorghum flour. In the sensory analysis, the authors observed that there was no significant difference in the preference for a specific pasta color. However, the samples developed with the BRS 305 (brown color and rich in tannins) were the least acceptable samples, receiving a lower acceptance for the flavor attribute with a higher astringency and a sandy sensation in the mouth. This could be related to the high tannin content of this sorghum genotype and the endosperm characteristic that was more farinaceous.

#### 3.2.5. Sorghum-Based Ingredients

The literature shows a high use of sorghum as flour for developing new products but also calls attention to the extraction of the starch from the grain, which is also an interesting ingredient due to its high content of resistant starch that can be used to formulate food products with less glycemic impact. This is because products with this sorghum starch present a high dietary fiber content and properties of slowly digestible starch without affecting the sensory attributes [60].

There has been an increase in research about sorghum grain extruded products (Figure 5), like snacks and breakfast cereals, along with extruded sorghum flour, which is used as an ingredient in cakes, breads and beverages. The use of the extruded flour instead of the raw sorghum flour can contribute to better digestion of sorghum. However, this process can reduce the content of some biocomponents that are interesting to human health [57,62,63]. According to Xu, Wang and Zhao [10], the extrusion process affected the total phenolic content and total flavonoids of sorghum, which are related to the loss of the biological functions of the phenolic components in sorghum. Other studies are needed to define the process conditions that maintain the phenolic content and its biological activity and, also, processes that help reduce the anti-nutritional factors in sorghum, improving nutrient digestion.

## 4. Conclusions and Future Directions

The data presented in this study contribute to highlight the potential use of sorghum in daily meals and food products for both homemade and industrial processing, contributing to the valorization and insertion of sorghum in human food. However, further studies are required on home methods of processing sorghum grains to show the feasibility in terms of grain yield, the practicality in the methods analyzed and the acceptance of the preparation by the consumer.

This study also provides an overview of the various food products developed from sorghum in the last decade, including GF bakery and pasta products, which are essential to provide quality food to consumers who need or choose a gluten-free diet. However, studies on commercial sorghum-based food products are still scarce, and further research is required to investigate the industrial use of sorghum from different countries as well as to evaluate the presence of GC and GF versions and the nutritional profile of these sorghum-based products.

## Figures and Tables

**Table 1:** Studies about sorghum-based products and their principal findings on physical properties, nutritional characteristics and/or sensory acceptance.

Sorghum-Based Product		Key Findings	Reference
Beverages	Powdered drink mix using extruded tannin-sorghum flour and extruded tannin-free sorghum flour.	- Both products had low lipid and high fiber and protein contents. - Both products were accepted by consumers in relation to color, flavor, texture, aroma and overall acceptability. - Tannin presence did not negatively influence the acceptance of the drinks.	Queiroz et al. [18]
	Sweet-sorghum juice treated with microbial and enzymatic bioprocessing.	- The use of microbial and enzymatic bioprocessing was positive to the sorghum juice development. - Product feasible to be used to develop new plant-based functional products.	Sharma et al. [41]
	Sorghum malted beer(40, 60 and 100% of SF).	- The sorghum beer presented differences of color, acidity and flavor when compared with the control sample (barley beer). - Second organoleptic evaluation, the beer prepared with 60% of malted sorghum was comparable to the barley beer.	Davana and Revanna [22]
Porridges	Sorghum porridges containing 4, 8 and 10% of solids.	- Sorghum porridges containing 8–10% of solids were described as thick. - Not indicated for consumption by children.	Makame et al. [42]
	Microwave treatment of sorghum to improve porridges development.	- Use of treated grains improved the flour stability. - Porridges were less rancid and less viscous. - More suitable for infant consumption.	Adebowale et al. [43]
Bakery products	GFB developed SF (BRS501—white sorghum (WS) and BRS332—bronze sorghum (BS)) with potato starch.	- It was possible to make GFB with 75% WS and 100% BS without affecting the sensory acceptance. - It was noted that a soft crumb to the GFB samples developed with 100% BS when compared with samples containing 50% BS. - GFB developed with 100% WS presented a higher crumb firmness, and a water adjustment was necessary to obtain samples with a soft crumb.	Centeno et al. [14]
	Sourdough GFB developed with SF with legume flour (cowpea and chickpea flour)	- GFB containing legumes, especially the chickpea, showed a high acceptability. - GFB developed with 60% SF + 10% chickpea flour + 30% corn starch showed best textural, nutritional and sensorial characteristics.	Olojede, Sanni and Banwo [44]
	GFB developed with SF combined with starch and hydrocolloids	- GFB containing 90% SF + 10% RF + 3% xantham gum presented the best sensory attributes and a higher fiber content than GFB formulated with potato starch + xantham gum or tapioca starch + HPMC.	Akin et al. [45]
	GF chocolate cakes developed with SF or Teff	- SF and Teff caused an increase in fiber content. - No changes in overall acceptance were observed in GF cakes containing SF or Teff. - GF cakes containing SF showed greater sensory acceptance for color, aroma and texture attributes.	Nespeca et al. [46]
	Madeleines (Spanish cake)	- Hardness and gumminess were lower in sorghum formulation. - Madeleines developed with 35,6% of SF combined with wheat flour showed suitable organoleptic and textural properties, being similar to the 100% wheat madeleines.	Moreno et al. [47]
	GF cookies developed with 100% SF or combined with chia (CF) (50% SF + 50% CF)	- 100% SF cookies had a lower sensory acceptance than 50% SF + 50% CF cookies. - Combination of chia with sorghum flour had a positive effect on the texture and flavor sensory attributes.	Bôa et al. [48]
	GF cookies prepared with 50% SF + 50% Turkish beans (TBF)	- Cookies had a good acceptance from the technological and sensory characteristics. - No hydrocolloid addition was necessary.	Shahzad et al. [49]
Pasta products	GF pasta developed with brown, red and white SF	- GF pasta containing red SF presented a similar sensory acceptance with the control sample (pasta made with RF). - GF pasta prepared with BRS305 (brown SF) presented a lower flavor acceptance. - Brown color, whole-grain flavor, whole-grain odor, sandy surface and total condensed tannins were related to a negative effect on sensory acceptance.	Oliveira et al. [50]
	Dry white Chinese noodles	- SF addition affected the pasting properties. - Adding more than 10% of SF could have an adverse effect on physical properties. - Noodles with 30% of SF could be produced without color impact but with minor negative effect on texture.	Xu et al. [51]
Sorghum-based ingredients	Extruded ready-to-eat cereals	- Non-tannin sorghum has potential to develop breakfast cereals with minimal impact on nutritional profile or sensory properties.	Mkandawire et al. [35]
	Sorghum expanded extrudates	- Sorghum tannins had the highest antioxidant capacity but lowest values of sensory acceptability. - White sorghum extrudates presented the higher expansion and sensory acceptance.	Chávez et al. [52]
	Cream cheese added with raw or extruded SF	- Cream cheese with SF (raw or extruded) presented a similar sensory acceptance and purchase intention of the cream cheese with no SF added. - Cream cheese with SF (raw or extruded) presented a higher protein content. - Raw flour presented a better sensory acceptance than the extruded flour.	Correia et al. [53]

## Data Availability

The data presented in this study are available on request from the corresponding author.
